# Diagnosis and treatment of uterine sarcoma: A multicenter, real-world study in western China

**DOI:** 10.1097/MD.0000000000028220

**Published:** 2021-12-23

**Authors:** Kemin Li, Rutie Yin, Li Li, Danqing Wang, Li Li, Cailing Ma, Qianchuan Ren, Guqing Wang, Yang Fan, Honggui Zhou, Zi Liu, Tao Li, Kunrong Luo, Dingqing Kui, Jingyi Wang

**Affiliations:** a The Department of Obstetrics and Gynecology, West China Second University Hospital of Sichuan University, Chengdu, Sichuan, China; b Key Laboratory of Birth Defects and Related Diseases of Women and Children (Sichuan University), Ministry of Education, Chengdu, China; c Hospital Affiliated to Guangxi Medical University, Nanling, China; d Tumor Hospital Affiliated to Xinjiang Medical University, Xingjiang, China; e The First Affiliated Hospital of Xinjiang Medical University, Xingjiang, China; f The Affiliated Hospital of Southwest Medical University, Luozhou, China; g Shaanxi Provincial Cancer Hospital, Xian, China; h NingXia people's Hospital, Yinchuan, China; i Affiliated Hospital of North Sichuan Medical College, Sichuan, China; j The First Affiliated Hospital of Xi’an Jiaotong University, Xian, China; k The Third People‘s Hospital of Chengdu, Chengdu, Sichuan, China; l The Second Hospital of Liangshan Yi State, Sichuan Province, Sichuan, China; m Dazhou Central Hospital, Sichuan Province, Sichuan, China; n Department of Obstetric and Gynecology, The Second Affiliate Hospital of Chengdu Medical College, China National Corporation 416 Hospital, Sichuan, China.

**Keywords:** multicenter, real-world study, uterine sarcoma, western China

## Abstract

A detailed understanding of the diagnosis and treatment of uterine sarcoma in the real world is required due to its low incidence, high malignancy, lack of specific symptoms, and lack of high-level evidence supporting its clinical diagnosis and treatment. This study aimed to provide a basis for the standardized diagnosis and treatment of uterine sarcoma. It retrospectively analyzed the real-world data on the diagnosis, treatment, and prognosis of uterine sarcoma in western China.

The clinical and pathological data of patients with uterine sarcoma diagnosed and treated between January 2009 and January 2019 in 13 medical centers from 4 western provinces of China, Sichuan, Guangxi, Shaanxi, and Xinjiang, were collected and further examined by univariate and multivariate analyses to find possible risk factors affecting the prognosis of uterine sarcoma.

A total of 299 patients with various pathological types of uterine sarcoma were included, with an average age of 47.7 ± 11.1 years. The univariate and multivariate analyses showed that age (*P* = .0081), family history (*P* = .0358), and chemotherapy regimen (*P* = .0005) significantly correlated with progression-free survival; while age (*P* = .0393) and International Federation of Gynecology and Obstetrics staging (*P* = .0141) significantly correlated with overall survival.

As age increased, the risk of death in patients with uterine sarcoma increased; The disease tended to progress faster in lower-age patients. A family history of tumors had an impact on disease progression; however, the way in which it affected needs further exploration. Different chemotherapy regimens affected the patient's disease progression. This study suggested that the anthracycline chemotherapy regimen was slightly better.

## 1 Introduction

Uterine sarcoma accounts for about 1% of all female genital tract malignant tumors and 3% to 7% of malignant tumors in the body of uterus.^[1]^ However, its etiology remains elusive. Studies showed that the long-term use of tamoxifen resulted in 3 fold increased risk of uterine sarcoma; patients undergoing pelvic radiotherapy might develop uterine sarcoma in the long term.^[2,3]^ It is difficult to clarify the nature of tumors preoperatively by imaging tests. Hence, patients with uterine sarcoma were mostly diagnosed with benign tumors of the uterus before surgery, and the diagnosis of uterine sarcoma could only be made by postoperative pathological examination. A detailed understanding of the diagnosis and treatment of uterine sarcoma in the real world is required due to its low incidence, high malignancy, lack of specific symptoms, and lack of high-level evidence supporting its clinical diagnosis and treatment. Also, more effective treatment options need to be explored, providing references for the diagnosis and treatment of uterine sarcoma.

Most studies on uterine sarcoma have been retrospective, small-sample studies, leaving various problems unsolved yet. Western China is a densely populated, economically backward, and vast region in China. We summarized the diagnosis and treatment of uterine sarcoma in 13 medical centers from 4 central and western provinces over the past 10 years in the real world to standardize and explore the diagnosis and treatment of uterine sarcoma, with a view to performing statistical analysis on real-world data and hoping to provide reference for clinically unresolved or controversial issues.

## 2 Research method

### 2.1 Research participants

The research participants were 299 patients with uterine sarcoma diagnosed and treated in the 13 medical centers in Sichuan, Guangxi, Shaanxi, and Xinjiang for the past 10 years (January 2009–January 2019) in western China.

The data were basic clinical information of the research participants collected from the electronic health record system of each center, including age, body mass index, diagnostic method [diagnosis and curettage (D&C), hysteroscopy and surgical pathology], a family history of malignant tumors, comorbidity (hypertension, diabetes, etc.), tumor markers (CA125, carcinoembryonic antigen [CEA], carbohydrate antigen199 [CA199], carbohydrate antigen153 [CA153], and human epididymis protein 4 [HE4]), surgical approach (laparotomy and laparoscopy), intraoperative bleeding (mL), intraoperative injury, surgical outcome (R0, R1, and R2), pathological type, the International Federation of Gynecology and Obstetrics (FIGO) staging, time from the first chemotherapy to surgery (minutes), chemotherapy regimen (anthracycline chemotherapy and nonanthracycline chemotherapy), radiotherapy, and so forth.

Patients with sarcomas not originating in the uterus, uterine sarcomas combined with other types of malignant tumors, or malignant tumors complicated by other systems were excluded from this study, while patients with undetermined pathological types of uterine sarcomas were included in the study.

The study was approvalled Medical Ethics Committee of West China Second University Hospital, Sichuan University (Ethical Lot Number: 20200076) and No need for informed consent.

### 2.2 Diagnosis and treatment

The research participants were diagnosed using 3 approaches.

1. D&C. Uterine sarcoma was pathologically diagnosed based on the scraped endometrial tissue inside the uterus using the D&C procedure.
2. Hysteroscopy. Uterine sarcoma was pathologically diagnosed based on partially removed endometrial or lesion tissue using hysteroscopy.
3. Patients who were preoperatively diagnosed with uterine fibroids or other benign diseases but pathologically diagnosed with uterine sarcoma after surgical treatment.

The surgical approaches were divided into laparotomy and laparoscopy. The postoperative chemotherapy regimens varied significantly among centers. Based on whether the chemotherapy regimens contained anthracyclines or platinum-based drugs, the regimens were divided into no chemotherapy, anthracycline-containing chemotherapy regimen, platinum-based chemotherapy regimen, anthracycline-platinum combined chemotherapy regimen, and others. Based on the status of residual tumor after surgery, the surgical outcomes were divided into R0 (no residual tumor), R1 (residual tumor diameter <1 cm), R2 (1 cm <residual tumor diameter <2 cm), and unsatisfactory surgical cytoreduction. Based on the scope, surgery was divided into standard surgery (radical hysterectomy + bilateral salpingo-oophorectomy ± pelvic lymph node dissection) and nonstandard surgery.

### 2.3 Research endpoint

The primary study endpoints were progression-free survival (PFS) and overall survival (OS).

### 2.4 Statistical analysis

SAS 9.1 was used for data analysis. Descriptive analysis was performed on the patients’ demographic information and preoperative conditions. Multiple imputation was used to fill in missing values for continuous variables, while the mean value was used for unsatisfactory convergence and the endpoints were not filled in. The stepwise strategy was used for variable selection. The missing data were summarized using PROC MIANALYZE. Cox regression analysis was performed to investigate the risk factors related to the prognosis of uterine sarcoma. The Kaplan–Meier method was used to conduct survival analysis and draw survival curves. A P value <.05 indicated a statistically significant difference.

## 3 Results

### 3.1 Basic characteristics of research participants

The basic characteristics of the research participants are shown in Table 1. A total of 299 participants, with an average age of 47.7 ± 11.1 (14–76) years, from the 13 medical centers meeting the criteria were included. These included 119 patients with endometrial stromal sarcoma (ESS, 39.8%), 86 with uterine leiomyosarcoma (uLMS, 28.8%), 67 with uterine carcinosarcoma (22.4%), 21 with uterine adenosarcoma (7.0%), and 6 (2%) others. Further, the study included 119 patients with stage I (39.8%), 26 with stage II (8.7%), 46 with stage III (15.4%), and 22 with stage IV (7.4%)according to 2009 FIGO staging; the remaining 86 patients could not be staged due to nonstandard surgery (28.8%).

**Table 1:** The basic characteristics of participants.

Variety	Results	Variety	Results
Yr (N)	299	Diagnosis method, n (%)	150
Mean (SD)	47.7 (11.08)	Hysteroscope	36 (24.0)
Median (Q1;Q3)	48 (41;54)	Curettage	59 (39.3)
Min; Max	14;76	Surgical examination	55 (36.7)
BMI (N)	289	Pathological type n (%)	299
Mean (SD)	22.7 (3.75)	Rhabdomyosarcoma	4 (1.3)
Median (Q1;Q3)	22 (20;25)	Myxofibrosacroma	2 (0.7)
Min; Max	14;38	Uterine carcinosarcoma	67 (22.4)
CA125 (N)	176	Endometrial stromal sarcoma	119 (39.8)
Mean (SD)	85.0 (208.5)	Uterine leiomyosarcoma	86 (28.8)
Median (Q1;Q3)	35 (35;37)	Uterine gland sarcoma	21 (7.0)
Min; Max	35;2341	FIGO stage n (%)	213
CEA (N)	174	I	119 (55.9)
Mean (SD)	6.7 (17.01)	II	26 (12.2)
Median (Q1;Q3)	5 (5;5)	III	46 (21.6)
Min; Max	5;224.8	IV	22 (10.3)
CA199 (N)	176	Family history of cancer, n (%)	299
Mean (SD)	72.4 (420.21)	yes	20 (6.7)
Median (Q1;Q3)	31 (30.9;30.9)	no	279 (93.3)
Min; Max	0;5508.6	Complications, n (%)	299
CA153 (N)	170	yes	70 (23.4)
Mean (SD)	21.6 (21.06)	no	229 (76.6)
Median (Q1;Q3)	25 (11;25)	Chemotherapy n (%)	299
Min; Max	2.3;218.2	no	67 (22.4)
HE4 (N)	124	Anthracylines	32 (10.7)
Mean (SD)	189.6 (690.44)	Platinum	65 (21.7)
Median (Q1;Q3)	140 (139;140)	Anthracylines + Platinum	125 (41.8)
Min; Max	29.7;7780.0	Others	10 (3.3)

BMI = body mass index, CA125 = carbohydrate antigen125, CEA = carcinoembryonic antigen, CA199 = carbohydrate antigen199, CA153 = carbohydrate antigen153, HE4 = human epididymis protein 4, FIGO = the International Federation of Gynecology and Obstetrics, PFS = progression-free survival, OS = overall survival.

A total of 150 patients had a clear record of diagnostic methods, whereas the other 149 patients had no such records. Among these, 59 patients underwent D&C; the scraped endometrial tissues were sent for pathological examination and were diagnosed with uterine sarcoma (39.3%). Further, 36 patients underwent hysteroscopy; the partially removed endometrial or lesion tissues were sent for pathological examination and were diagnosed with uterine sarcoma (24%). Also, 55 patients underwent surgery for uterine fibroids or other diseases, while pathological examinations unexpectedly revealed uterine sarcoma (36.7%). Among the 299 patients, 67 received no postoperative chemotherapy (22.4%), 32 received anthracycline chemotherapy regimen (10.7%), 65 received platinum-based chemotherapy (21.7%), 125 received anthracycline-platinum combined chemotherapy (41.8%), and 10 patients were treated with other chemotherapy regimens (3.3%).

Moreover, 20 patients had a definite family history of tumor, and 70 patients had various types of comorbidities, primarily hypertension and diabetes.

### 3.2 Risk factors related to patient's prognosis

The risk factors related to the patient's prognosis are detailed in Table 2. The univariate analysis showed that body mass index (*P* = .5332), time from the first chemotherapy to surgery (*P* = .6940), CA125 (*P* = .4942), CEA (*P* = .2018), CA199 (*P* = .3808), CA153 (*P* = .7911), HE4 (*P* = .7407), surgical approach (*P* = .7650), surgical scope (*P* = .5526), intraoperative injury (*P* = .1148), and postoperative pathology (*P* = .4992) had no significant correlation with PFS. A family history of tumor (*P* = .6327), comorbidity (*P* = .8945), time from the first chemotherapy to surgery (*P* = .1195), CA125 (*P* = .4955), CEA (*P* = .9188), CA199 (*P* = .2455), CA153 (*P* = .8042), HE4 (*P* = .2003), surgical approach (*P* = .9144), surgical scope (*P* = .2822), intraoperative bleeding (*P* = .2498), intraoperative injury (*P* = .3038), postoperative pathology (*P* = .7525), chemotherapy regimen (*P* = .7145), and radiotherapy (*P* = .5100)did not significantly correlate with overall survival (OS). The multivariate analysis revealed that age (*P* = .0081), family history of tumor (*P* = .0358), and chemotherapy regimen (*P* = .0005) significantly correlated with PFS; while age (*P* = .0393) and FIGO staging (*P* = .0141) significantly correlated with OS.

**Table 2:** Risk factors related to prognosis.

POINT	Variety	Estimate	ERR	*P* value	HR [95% CI]
PFS	Yr	−0.04521	0.01708	.0081	0.956[0.924,0.988]
	Family history	−1.25971	0.60002	.0358	0.284 [0.088,0.920]
	Chemotherapy	−0.40634	0.11711	.0005	0.666 [0.529,0.838]
	FIGO stage	0.16314	0.09671	.0916	1.177 [0.974,1.423]
OS	Yr	0.08570	0.04158	.0393	1.089 [1.084, 1.182]
	FIGO stage	0.62884	0.25605	.0141	1.875 [1.135, 3.098]

FIGO = the International Federation of Gynecology and Obstetrics, PFS = progression-free survival, OS = overall survival.

### 3.3 Survival analysis

**Figure 1:**
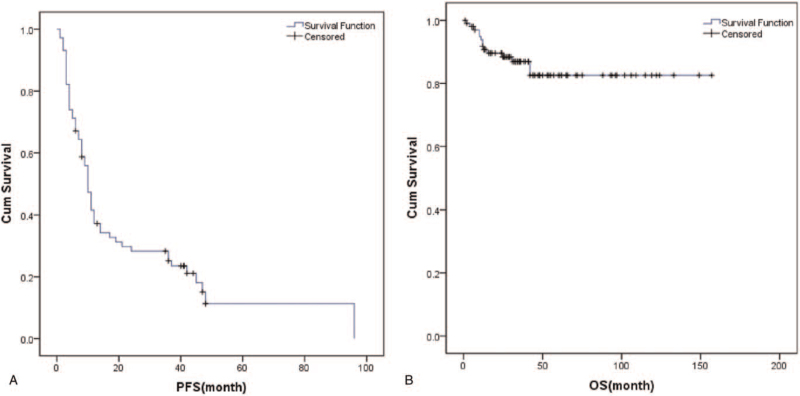
Survival curve (A: PFS, B: OS).

The patients’ survival curves are shown in Figure 1. A total of 59 patients had follow-up PFS data [median PFS 10.0 months, 95% confidence interval (CI): 4.0–37.0]. Further, 14 patients had follow-up OS data; the time of death was 2 to 42 months after the end of first-line treatment. However, the median OS could not be estimated due to a large number of censored persons.

## 4 Discussion

The incidence of uterine sarcoma is low, accounting for about 3% to 7% of all malignant tumors of the uterus, including ESS, unexpected uterine sarcoma, and uLMS. Tumor staging is the most important prognostic factor.^[4]^ A systematic review of data from 1970 to 2011 was reported in the United States in 2012. The results showed that uLMS was the most common subtype (63%), followed by ESS (21%).^[5]^ Adenosarcoma, rhabdomyosarcoma, and perivascular epithelioid cell tumor are rare.^[6]^ No large-sample reports are available on uterine sarcoma in China yet.

Uterine sarcoma mostly occurs in women experiencing perimenopause or postmenopause. Moreover, different pathological types occur. Leiomyosarcoma occurs primarily in women aged more than 40 years. Low-grade ESS mostly occurs in the age group of 40 to 55 years. Adenosarcoma primarily occurs in postmenopausal women (aged 58 years on average), but it may also occur in adolescents or young women (30%). The incidence of carcinosarcoma and adenosarcomais significantly higher than that in patients with other types of uterine sarcoma.^[3,7]^ Studies showed that age was a risk factor affecting the prognosis of uterine sarcoma.^[7–10]^ Burghaus et al^[8]^ analyzed the prognostic factors in 145 patients with uterine sarcoma, including 49 with malignant mixed Mullerian tumor, 23 with ESS, 57 with uLMS, and 14 with endometrial sarcoma. The results of multivariate analysis indicated that patient's age, FIGO staging, smoking history, and pelvic radiotherapy were associated with PFS and thus were the independent risk factors for PFS. As age increased, the risk of death increased (hazard ratio [HR] = 1.07; 95% CI 1.03–1.10; *P* < .001). Brandon-Luke et al^[9]^ investigated the prognostic factors of 2414 patients with low-grade ESS and 1383 patients with high-grade ESS. The multivariate analysis demonstrated age as a factor affecting the prognosis of patients with low-grade ESS; the risk of death increased with the increase in age. Zhang^[10,11]^ studied the prognostic factors in 132 patients with uterine sarcoma, including 104 with low-grade ESS and 28 with carcinosarcoma. The results showed that age was an independent risk factor for PFS and OS in patients with uterine sarcoma. The present study used Cox regression analysis to study the relationship between the patient's age and prognosis and reached a conclusion consistent with what was reported, with a positive predictive value; the HR and 95% CI were 1.089 and (1.084–1.182), respectively. The results showed that the patient's age was a risk factor for prognosis. The risk of death increased with age of patients. However, the disease progressed faster in lower-age patients. Hence, it was recommended that active strengthened adjuvant treatments should be given when necessary. However, it was impossible to accurately estimate the age threshold due to a large amount of censored data in this study; also, the study did not clarify the possible reasons for the faster progression in lower-age patients, which required further investigation.

A family history of a tumor is an essential tool for malignant tumor risk assessment. It can help with an individual's risk assessment of primary cancer, recommendations for the tumor screening of family members, and referral for genetic counseling and genetic testing.^[12]^ Whether the family history of malignant tumor affects the prognosis of uterine sarcoma remains unclear. Our team attempted to retrieve relevant studies or data from databases such as PubMed and Ovid, but with no success. In this study, the Cox multivariate analysis revealed that the family history of the tumor impacted disease progression. However, the sample size was small, and the rate of loss to follow-up was high. Hence, large-sample, high-quality studies are needed for verification, especially regarding the impact mode.

Studies showed that the prognosis of different pathological types of uterine sarcoma varied a lot.^[3,5,7,13]^ In the case of no muscle infiltration or excessive hyperplasia of sarcoma, the prognosis of adenosarcoma was much better than that of carcinosarcoma. Low-grade ESS grew slowly and the prognosis was good, with a 5-year survival rate of 90% for stages I and II and 50% for stages III and IV. In contrast, the prognosis of an early-stage leiomyosarcoma was poor, with a recurrence rate of 53% to 71%, a 5-year survival rate of only 15% to 25%, and a median survival of 10 months. High-grade ESS had a high recurrence rate, and undifferentiated endometrial sarcoma resulted in a poor prognosis. In this study, the Cox regression analysis did not reveal a significant correlation between tumor pathological type and patient's prognosis, which might be related to the loss of follow-up for most patients. In the National Comprehensive Cancer Network Clinical Practice Guidelines in Oncology, version 1, 2020, molecular classification has been adopted for endometrial cancer. A suitable molecular classification for uterine sarcoma that can help in prognosis and selection of adjuvant therapy is worth exploring.

Chemotherapy still plays a critical role in the adjuvant treatment of uterine sarcoma. Various studies confirmed that postoperative adjuvant chemotherapy could significantly improve the PFS and OS of patients.^[9,14]^ The National Comprehensive Cancer Network and FIGO guidelines^[3,15]^ recommended active chemotherapy for patients with poor prognostic pathological types of cancer, such as high-grade ESS and uterine carcinosarcoma, since it could significantly improve the prognosis of patients, suggesting combined chemotherapy regimens containing anthracyclines or platinum. In this study, the patients were divided into groups based on whether the chemotherapy regimens contained anthracycline or platinum. The multivariate analysis showed that chemotherapy could significantly improve the prognosis of patients, and the anthracycline chemotherapy regimen was slightly better.

Although 299 patients with uterine sarcoma were included in this study, the number of patients for each pathological type varied a lot, and the treatment options in different medical centers were quite different too. In particular, the large difference in selected chemotherapy regimens and the large number of censored data of patients seriously affected the prognostic factor analysis in this study; therefore, PFS and OS could not be estimated accurately. This study has obvious shortcomings due to the influence of the aforementioned confounding factors. Nevertheless, it was a real-world study of 4 major medical centers in western China (Sichuan, Guangxi, Shaanxi, and Xinjiang). Still, it had a specific reference value for the clinical diagnosis and treatment of uterine sarcoma. At the same time, it was suggested to improve communications with patients and increase the follow-up rate. We look forward to large-sample, high-quality studies in the future.

Approval Medical Ethics Committee of West China Second University Hospital, Sichuan University (Ethical Lot Number: 20200076).

## Author contributions

**Conceptualization:** Kemin Li, Rutie Yin, Li Li, Danqing Wang, Li Li, Cailing Ma, Qianchuan Ren, Guqing Wang, Yang Fan, Honggui Zhou, Zi Liu, Tao Li, Kunrong Luo, Dingqing Kui, Jingyi Wang.

**Data curation:** Kemin Li, Rutie Yin, Li Li, Danqing Wang, Li Li, Cailing Ma, Qianchuan Ren, Guqing Wang, Yang Fan, Honggui Zhou, Zi Liu, Tao Li, Kunrong Luo, Dingqing Kui, Jingyi Wang.

**Formal analysis:** Kemin Li, Rutie Yin, Li Li, Danqing Wang.

**Funding acquisition:** Rutie Yin.

**Investigation:** Kemin Li, Rutie Yin, Li Li.

**Methodology:** Kemin Li, Rutie Yin, Li Li, Danqing Wang.

**Project administration:** Kemin Li, Rutie Yin.

**Resources:** Kemin Li, Rutie Yin, Li Li.

**Software:** Kemin Li.

**Supervision:** Kemin Li, Rutie Yin.

**Validation:** Kemin Li, Rutie Yin.

**Visualization:** Kemin Li, Rutie Yin.

**Writing – original draft:** Kemin Li, Rutie Yin, Li Li.

**Writing – review & editing:** Kemin Li, Rutie Yin, Li Li.
